# Built for success: Distribution, morphology, ecology and life history of the world's skinks

**DOI:** 10.1002/ece3.10791

**Published:** 2023-12-12

**Authors:** David G. Chapple, Alex Slavenko, Reid Tingley, Jules E. Farquhar, Marco Camaiti, Uri Roll, Shai Meiri

**Affiliations:** ^1^ School of Biological Sciences Monash University Clayton Victoria Australia; ^2^ CESAR Australia Brunswick Victoria Australia; ^3^ Mitrani Department of Desert Ecology, The Jacob Blaustein Institutes for Desert Research Ben Gurion University Midreshet Ben Gurion Israel; ^4^ School of Zoology & Steinhardt Museum of Natural History Tel Aviv University Tel Aviv Israel

**Keywords:** ecology, geographic range, life history, lizard, reproduction, Scincidae, thermal biology

## Abstract

In animals, the success of particular lineages can be measured in terms of their number of species, the extent of their geographic range, the breadth of their habitats and ecological niches, and the diversity of their morphological and life‐history traits. Here, we review the distribution, ecology, morphology and life history of skinks, a diverse lineage of terrestrial vertebrates. We compared key traits between the three subfamilies of skinks, and between skinks and non‐scincid lizards. There are currently 1743 described species of skink, which represent 24% of global lizard diversity. Since 2010, 16% of lizard descriptions have been of skinks. The centres of skink diversity are in Australia, New Guinea, southeast Asia, Oceania, Madagascar and central Africa. Compared with non‐scincid lizards, skinks have larger distributional ranges, but smaller body sizes. Sexual size dimorphism is rare in skinks. Almost a quarter (23%) of skinks exhibit limb reduction or loss, compared with just 3% of non‐scincid lizards. Skinks are more likely to be viviparous (34% of species) compared with non‐scincids (13%), and have higher clutch/litter sizes than non‐scincids. Although skinks mature later than non‐scincids, their longevity is similar to that exhibited by other lizard groups. Most skinks (88%) are active foragers, and they are more likely to be carnivorous than non‐scincids. Skinks are more likely to be diurnal or cathemeral than other lizard groups, but they generally have lower field body temperatures compared with non‐scincids. The success of skinks appears to be both a result of them hitting upon a winning body plan and ecology, and their capacity to regularly deviate from this body plan and adapt their ecology and life history (e.g. repeated limb reduction and loss, transitions to viviparity) to prevailing conditions.

## Graphical abstract

There are currently 1743 described species of skink, which represents 24% of global lizard diversity. The success of skinks appears to be both a result of them hitting upon a winning body plan and ecology, and their capacity to regularly deviate from this body plan and adapt their ecology and life history (e.g. repeated limb reduction and loss, transitions to viviparity) to prevailing conditions.

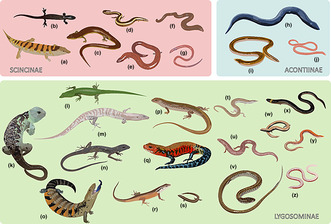

## 1 INTRODUCTION

What is success? For many faunal groups, one key measure of success is the number of extant species. The most diverse terrestrial vertebrate groups are non‐avian reptiles (Class Reptilia; 11,928 species, as at December 2022, Uetz et al., 2022) and birds (Class Aves; 11,161 species, as at July 2022, Handbook of the Birds of the World and Birdlife International, 2022), with relatively lower diversity evident in amphibians (Class Amphibia; 8523 species, as at October 2022, Frost, 2022) and mammals (Class Mammalia; 6495 species, as at April 2022, Mammal Diversity Database, 2022). The most speciose avian family are the tyrant‐flycatchers, with ~450 species (Handbook of the Birds of the World and Birdlife International, 2022). The most species‐rich mammalian family is the Muridae (murid rodents), with 843 species (Mammal Diversity Database, 2022), and the largest amphibian family is the Hylidae (tree frogs: 1036 species; Frost, 2022). However, the three most hyper‐diverse terrestrial vertebrate families are reptiles: colubrid snakes (Colubridae: 2088 species; ~50% of all snake species), skinks (Scincidae: ~1745 species) and geckos (Gekkonidae: 1542 species) (Meiri, 2020; Uetz et al., 2022).

Geckos are a cosmopolitan group whose success appears to be linked to the substantial variation in ecology, life history, reproduction and morphology that they exhibit (Meiri, 2020; Pianka & Vitt, 2003). As such, given this variation, it can be exceedingly difficult to identify the defining traits of geckos (Meiri, 2020). Similarly, colubrids make up ~50% of all snakes worldwide, have a near global distribution and exhibit extreme diversity in biological and ecological traits, to the extent that it is hard to make generalisations for the group (O'Shea, 2011, 2023; Pincheira‐Donoso et al., 2013; Uetz et al., 2022). However, while it appears that skinks may share similar signatures of success (e.g. cosmopolitan distribution, extreme variability in morphology and ecology) as geckos and colubrids, they are the most underappreciated lineage of the trio (Greer, 2007). Despite skinks making up a quarter of all lizard species (Uetz et al., 2022), their main centres of diversity (i.e. sub‐Saharan Africa, Madagascar, southeast Asia, the Indonesian Archipelago and Australasia; Chapple et al., 2021) fall outside of the major western science hubs of North America and Western Europe, where their diversity is very low, and they are often overlooked and understudied (Greer, 2007).

What is a skink? Skinks are a monophyletic lineage of lizards (Camaiti et al., 2022; Pyron et al., 2013; Tonini et al., 2016; Zheng & Wiens, 2016) that Greer (2007) defines based on a combination of five characters: (i) a characteristic pattern of plates in the composite osteoderm within each scale, (ii) a bony secondary palate, (iii) an open, rhomb‐like mesosternum (i.e. middle portion of the sternum) (except in very limb‐reduced lineages where it is closed), (iv) a distinctive tongue musculature (i.e. a longitudinal bundle of the genioglossus lateralis muscle running forward into the free part of the tongue parallel to the more medial hyoglossus muscle) and (v) the absence of a panting response (also see Pianka & Vitt, 2003). Skinks are the dominant members (~92% of described species) of the superfamily Scincomorpha (1886 species; Burbrink et al., 2020), which includes its closest relatives, the African spinytail lizards (Cordylidae, 68 species) and plated lizards (Gerrhousauridae, 38 species), and the North American night lizards (Xantusiidae, 37 species) (Uetz et al., 2022). Shea (2021) recently revised skink classification, recognising three subfamilies: Scincinae (typical skinks), Acontiinae (limbless skinks) and Lygosominae (lygosomine skinks). Shea (2021) also identified seven major lineages (or Tribes) within the Lygosominae: Ateuchosaurini (East Asian skinks), Eugongylini (eugongylin skinks), Lygosomini (lygosomin skinks), Mabuyini (mabuyin skinks), Ristellini (Indo‐Sri Lankan skinks), Sphenomorphini (sphenomorphin skinks) and Tiliquini (social skinks) (Uetz et al., 2022).

Skinks occur in almost all habitat types, from deserts to rainforests and from sea level to high elevation alpine areas above the tree line (the maximum altitude recorded is *Ablepharus ladacensis* at 5490 m elevation in the Himalayas; Greer, 2007; Pianka & Vitt, 2003). Most skink species are terrestrial, but the group encompasses many representatives that are fossorial, arboreal or semi‐aquatic (Meiri, 2018; Pianka & Vitt, 2003, and see below). Skinks come in a variety of shapes and sizes. Body size variation is substantial, with a 17‐fold variation in adult body length and an 1800‐fold difference in adult body mass (Greer, 2007; Meiri, 2018, see below). Skinks are the poster child for limb reduction and/or loss, evolving independently 53–71 times within the group (Camaiti et al., 2022)—more than in any other tetrapod clade. Likewise, it is estimated that there have been more independent evolutionary shifts from oviparity to viviparity in skinks than in any vertebrate group: at least 31 times (Blackburn, 1982, 1999, 2015). Skinks are also the only non‐mammalian amniote group that has converged on the ‘mammalian’ pattern of complex placentation and placentotrophy—which has occurred independently in six different skink groups (Blackburn, 2015; Griffith & Wagner, 2017). Interestingly, there have likely been four independent origins of the evolution of green blood pigmentation in skinks, unique among amniotes (Rodriguez et al., 2018). Skinks are the only reptile group apart from gekkotans and anoles to have independently evolved adhesive toepads (Williams & Peterson, 1982). Skinks also exhibit substantial variation in a range of ecological and life‐history traits, including activity times (most species are diurnal, but some are crepuscular or nocturnal; Pianka & Vitt, 2003; Slavenko et al., 2022), diet (most species are insectivorous, but some larger species are omnivorous or even herbivorous; Chapple, 2003) and sociality (several species, mostly in the Tribe Tiliquini, exhibit long‐term stable social aggregations; Chapple, 2003; Gardner et al., 2016; While et al., 2019).

While skinks have traditionally been understudied and neglected (Greer, 2007), the establishment of the International Union for Conservation of Nature (IUCN)'s Skink Specialist Group (https://www.skinks.org/) has been an important step in improving our understanding of their biology and ecology (e.g. Chapple et al., 2021). Here, we aim to provide a detailed synthesis of the distribution, morphology, ecology and life history of skinks worldwide and examine whether the key traits of skinks differ from other lizard groups.

## 2 MATERIALS AND METHODS

We gathered literature data on all recognised species of skinks (Appendices S1 and S2), based on the December 2022 edition of the Reptile Database (Uetz et al., 2022). We supplemented these with observations and measurements of skinks we took in the field and natural history museums. Description dates were obtained from Uetz et al. (2022). We follow the subfamily designations of Shea (2021) and recognise three monophyletic skink subfamilies: Scincinae, Lygosominae and Acontiinae.

Distribution data include point locality data and polygons, which were merged on a species basis, and point localities polygonised using alpha hulls (for species with >5 observations), minimum convex polygons (for species with 3–5 known localities) or buffered using a 1.78 km radius, as described in detail in Roll et al. (2017). Distribution data (from Caetano et al., 2022) are an update of the dataset used by Roll et al. (2017; internally named GARD 1.7).

Body size data were collected as snout‐vent length (SVL, in mm; Meiri, 2008), and then converted to mass (in g) using new allometric equations that consider leg development status (fully limed, limb‐reduced or having just one pair of limbs and limbless; Feldman et al., 2016; Meiri, 2010; Appendix S2) that we developed here (see below). This is because, for the same SVL, fully limbed skinks are much heavier than limbless species, with limb‐reduced species intermediate.

To do this, we collated a data set of literature data, our own measurements in the field and in the laboratory, and personal communication with colleagues, of skink SVL and mass data—provided both measurements are reported in the same publication for the same population (Appendix S2). When possible, we preferred data from males or post‐oviposition/post‐partum females (whichever had the largest sample size), to mixed samples of males and females. We did not include measurements of females known to be gravid or of hatchlings. When different measurements were reported in different works for the same species, we used the one with the largest sample. Our dataset contains mass and length data on 385 species: 11 acontiines (all limbless; 35% of acontiine species diversity), 323 lygosomines (2 limbless, 32 limb‐reduced, 289 fully legged; 27% of species) and 51 scincine species (7 limbless, 14 limb‐reduced and 30 fully limbed; 17% of species).

We log_10_ transformed mass and SVL data and tested for the effect of leg development and subfamily on their relationships. Following this, size was recorded as maximum SVL, because this is often the only type of datum available for many species, then converted to mass using the Equations (1), (2), (3), (4), above. Sexual dimorphism was calculated from mean SVL data of males and females (data from Liang et al., 2022). We used the Lovich and Gibbons ratio (Lovich & Gibbons, 1992; Smith, 1999), calculated as the SVL of the larger sex divided by the SVL of the smaller sex, minus one. This value is then multiplied by −1 for males to create a distribution that is symmetrical about zero.

Data on life‐history traits are an updated version of the dataset in Meiri (2018) (Appendix S1). Clutch size data are from Meiri, Feldman, et al. (2020) and Meiri, Avila, et al. (2020). Reproductive mode is treated as oviparous, viviparous (including ovoviviparous species), and mixed for species in which some females are egg laying and others give birth to live young (e.g. *Lerista bougainvillii*; Qualls & Shine, 1998). Age at first reproduction is the midpoint of maturity ages in months (for females, if data are reported separately for males and females). Longevity is the maximum reported value (in years; updated from Stark et al., 2020).

Microhabitats were categorised as arboreal, saxicolous, terrestrial, fossorial, semi aquatic, and their combinations for species frequently using more than one microhabitat (see Meiri, 2018; Appendix S1). Diet was treated as carnivorous if >90% of the reported food (by volume, if known) were animal matter (Appendix S1). Omnivores were considered to be species feeding predominantly on animals, but also including substantial amount of plants (10%–50% if numerical data were available). Species feeding mostly on plant matter (>50%) were considered herbivores. Foraging modes were classified as sit and wait, active foraging or mixed. Activity times were classified as nocturnal, diurnal or cathemeral for species that could (often) be found active during both night and day (Slavenko et al., 2022; Appendix S1). Field body temperatures (in °C) are tallied for active animals in the field and preferred body temperatures were taken from laboratory‐based studies in thermal gradients (Appendix S1).

For all quantitative traits that are calculated as means (i.e. all except body size and longevity, which are expressed as maxima), we averaged the smallest and largest reported means if more than one value was available. If no means were available, we averaged the smallest and largest observed values.

### 2.1 Analyses

All analyses were performed in R version 4.1.3 (R Core Team, 2022). Analyses are basic GLMs (ANCOVAs, with either normal or log_10_ error structures) or *chi*‐squared tests. Since we compared the three monophyletic skink subfamilies to each other, or skinks to all non‐skink lizards, phylogenetically informed analyses are irrelevant, and we did not use them. Thus, while conclusions regarding differences between subfamilies are valid, we do not infer them to mean that such differences evolve independently (Felsenstein, 1985), as they might represent some carry‐on effects of conserved ancestral trait states.

## 3 RESULTS

**FIGURE 1:**
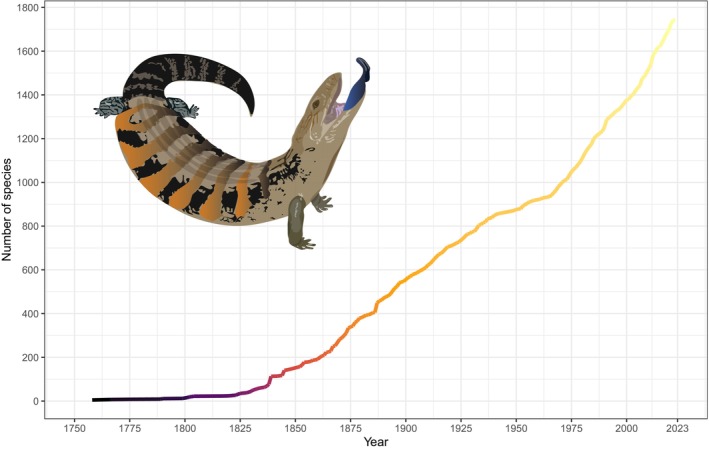
Growth in the number of described skink (Scincidae) species over time. Data from Uetz et al. (2022).

Skinks are a diverse lineage, with 1743 recognised species. This represents 23.8% of the 7310 recognised lizard species (as of December 2022; Uetz et al., 2022). Skinks continue to be described at a substantial rate, with ~20 new species per year during the last decade (Figure 1) resulting from the discovery and description of new species, and the splitting of species complexes. Since 2010, 16% of all new lizard species described have been skinks. Species diversity varies substantially among the subfamilies: Acontiinae (2 genera, 31 species), Lygosominae (133 genera, 1417 species) and Scincinae (33 genera, 294 species).

### 3.1 Distribution

**FIGURE 2:**
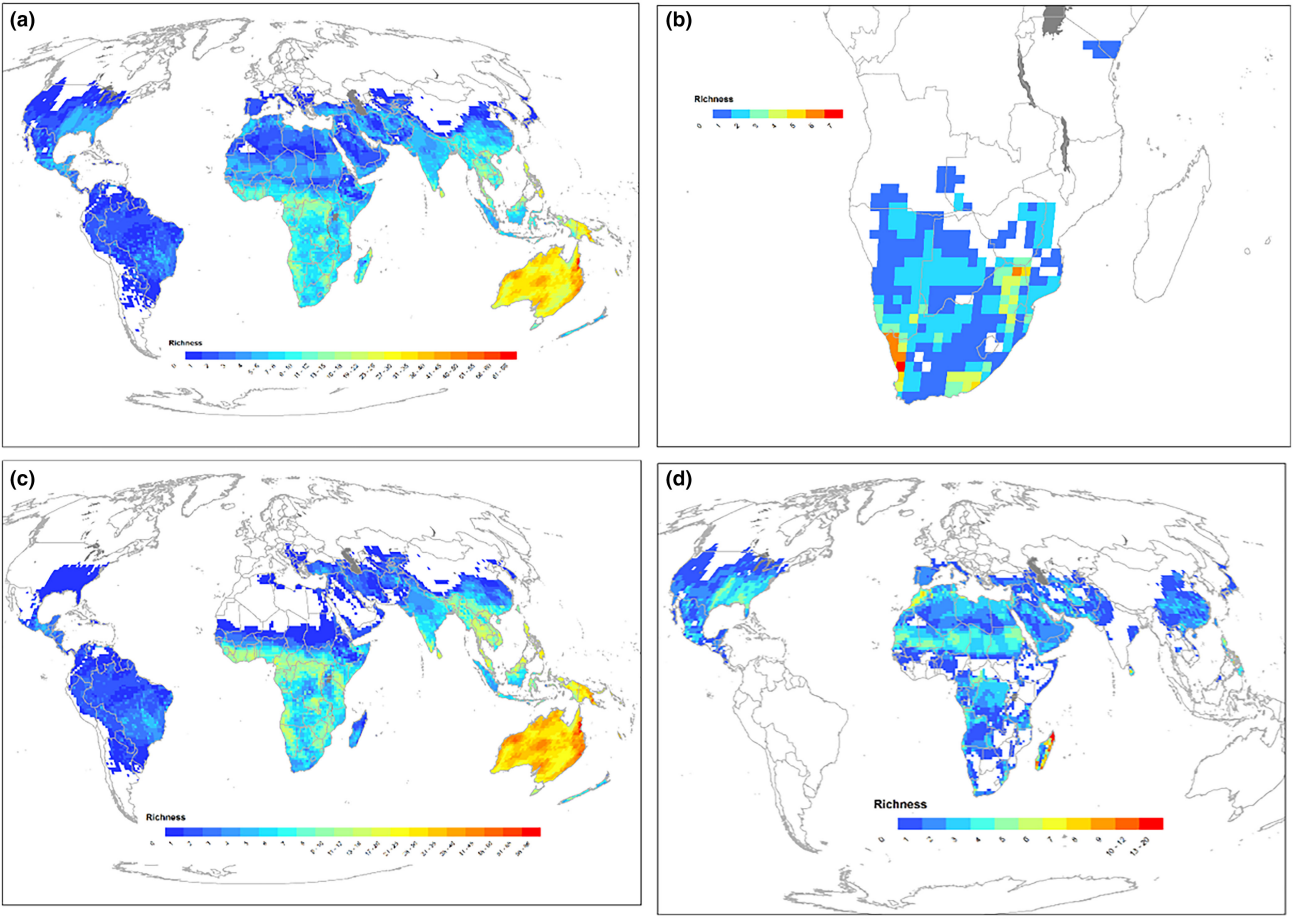
Species richness of skinks (Scincidae) (a) globally, (b) Acontiinae, (c) Lygosominae, (d) Scincinae. Data from GARD (http://www.gardinitiative.org/; version 1.7, see Caetano et al., 2022).

Skinks have a global distribution, occurring on every continent apart from Antarctica (Figure 2a). The centres of skink diversity are Australia, New Guinea, southeast Asia, Oceania (including New Zealand, New Caledonia and Pacific islands), Madagascar and southern and central Africa (Figure 2a). Relatively few skink species occur in the New World (North, Central and South America), and Europe (Figure 2a)—skink species comprise just 4% of the total lizard fauna of the Neotropics, 10% of the Nearctic and 14% of the Palaearctic fauna. In contrast, skink species make up 56% and 54% of the lizard fauna of Oceania and Australasia, respectively. The Acontiinae is endemic to southern Africa (Figure 2b). In contrast, the Lygosominae is nearly cosmopolitan, with a richness hotspot in Australia and New Guinea (Figure 2c). The Scincinae has a wide but patchy distribution in large parts of Asia, SW Europe and Africa, a wide distribution in North and central America and a richness hotspot in Madagascar (Figure 2d).

**FIGURE 3:**
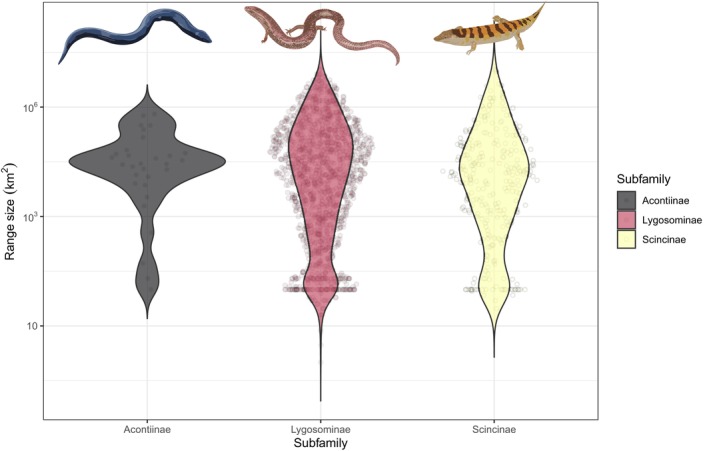
Violin plots comparing range sizes (log_10_ km^2^) among the three skink subfamilies. Data from GARD 1.7 (http://www.gardinitiative.org/; Caetano et al., 2022).

The geographical range sizes of skinks (8436 km^2^ ± 42.1 SD, log_10_ transformed, averaged and back transformed) are, on average, larger than those of non‐scincid lizards (5110 km^2^ ± 46.8 SD, *p* < .001). There are no significant differences in the range sizes among skink subfamilies (means ± SD, in km^2^, log_10_ transformed; Acontiinae: 4.20 ± 1.20, *n* = 30; Lygosominae: 3.94 ± 1.65, *n* = 1309; Scincinae: 3.82 ± 1.56, *n* = 285; *F* = 1.08, *p* = .34) (Figure 3).

### 3.2 Morphology

We found (Figure S1) that fully limbed lygosomines and scincines were heavier than limb‐reduced ones (limb reduction definition follows Camaiti et al., 2022), for a given SVL, but there were no differences between the subfamilies (and within leg development modes—no interactions). Limbless acontiines, however, were lighter than limbless scincines (controlling for SVL). The two limbless lygosomines in our sample were closer to similar length scincines than to similar length acontiines (all acontiines are limbless). We thus use the following allometric equations to derive mass (in g) from snout‐vent length (in mm):

Fully limbed skinks (Lygosominae and Scincinae):

(1) $$\log_{10}\left(\text{mass}\right)=\log_{10}\left(\mathrm{SVL}\right)\times 3.139-4.951$$

Reduced‐limb skinks (Lygosominae and Scincinae):

(2) $$\log_{10}\left(\text{mass}\right)=\log_{10}\left(\mathrm{SVL}\right)\times 2.519-4.234$$

Limbless (Lygosominae and Scincinae):

(3) $$\log_{10}\left(\text{mass}\right)=\log_{10}\left(\mathrm{SVL}\right)\times 2.436-4.357$$

Acontiinae (all limbless):

(4) $$\log_{10}\left(\text{mass}\right)=\log_{10}\left(\mathrm{SVL}\right)\times 3.199-6.439$$

**FIGURE 4:**
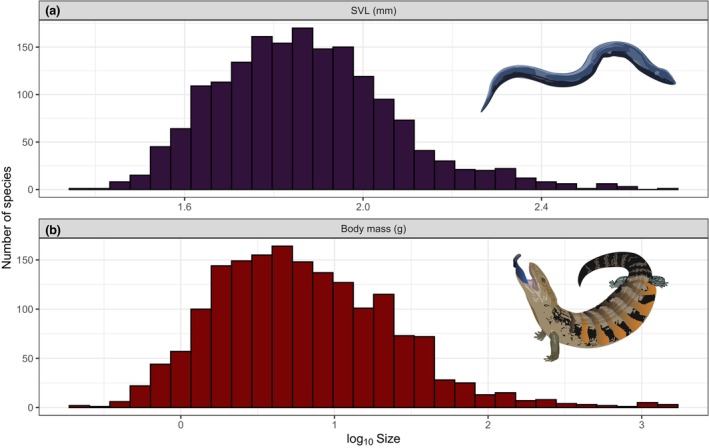
Frequency histogram of skink (Scincidae) body size: (a) snout‐vent length (log_10_ SVL), (b) mass (log_10_ g).

Skinks are relatively small lizards (Figure 4), with a shorter mean maximum SVL of 83.2 ± 46.6_SD_ mm; versus 99.6 ± 90.1 for non‐scincids (*t* = 7.25, *p* < .0001; skinks *n* = 1729, non‐scincids *n* = 5000) and lower mean maximum body mass (6.4 ± 3.8_SD_ g; vs. 10.9 ± 5.1 g for non‐scincids; *t* = 12.33, *p* < .0001). The smallest skink species is the limbless *Paracontias fasika*, with a max mass of 0.23 g, which is the 6th smallest lizard overall (max mass calculated from SVL; *Scincella macrotis* is 10th; 0.24 g). The largest skink, *Bellatorias major*, is only the 74th largest lizard overall (max mass 1537 g, calculated from SVL); *Tiliqua scincoides* and the extinct *Chioninia coctei* are tied in #77 (max 1405 g), *Tiliqua nigrolutea* is #87, *Corucia zebrata* and *T. rugosa* are tied in #99, and *T. gigas*, with a max weight of 1019 g, is the only other skink that can grow larger than 1 kg. The limbless *Acontias plumbeus* is the longest skink (#68 of all lizards; 500 mm SVL), *Bellatorias major* is the longest limbed skink (max 391 mm) and *Scincella macrotis* is the shortest (#22; max 24 mm SVL).

**FIGURE 5:**
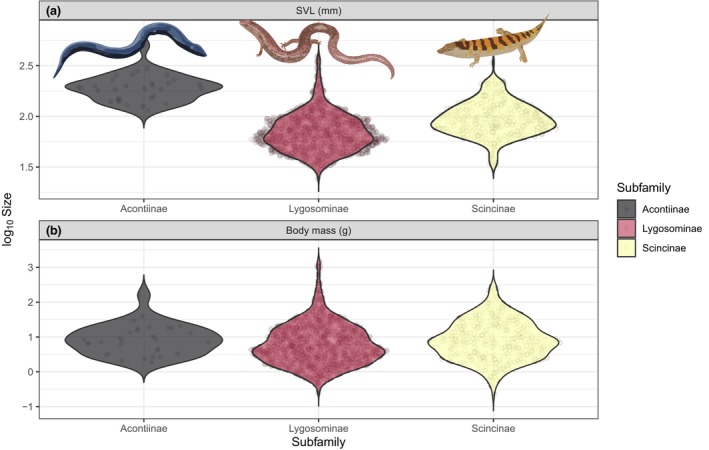
Violin plots comparing the (a) snout–vent length (log_10_ mm) and (b) mass (log_10_ g) of skink subfamilies.

In terms of length variation among the skink subfamilies, Acontiinae (mean 201 ± 72_SD_ mm, *n* = 31) comprises, on average, of the longest species, Lygosominae (mean 77 ± 42_SD_ mm, *n* = 1408) of the shortest, and Scincinae (mean 102 ± 42 mm, *n* = 290) is intermediate (*F* = 159.6, *p* < .0001; Figure 5a). Similarly, for mass, Acontiinae species are slightly heavier (mean of mass logarithms, back transformed: 8.3 ± 2.7 g), then Scincinae (mean 8.0 ± 3.6 g) and lygosomines are the lightest (6.0 ± 3.8 g; though only the difference between lygosomines and scincines is significant, *t* = 3.43, *p* = .0006; Figure 5b).

**FIGURE 6:**
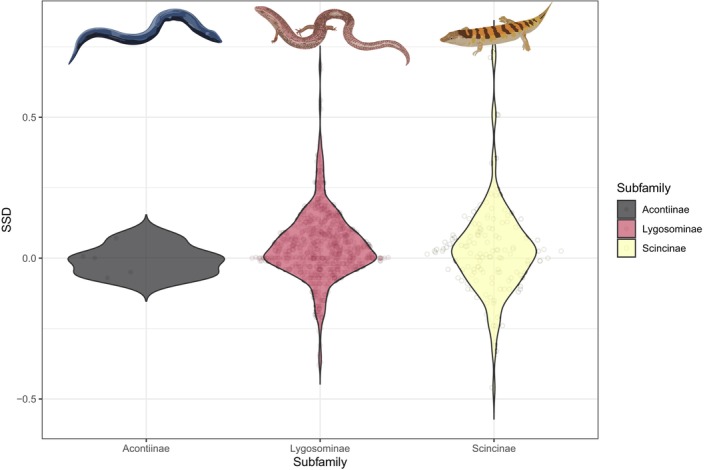
Violin plots comparing sexual size dimorphism (SSD, based on maximum snout‐vent length) for skink subfamilies. SSDs represented by Gibbons and Lovich (1990) ratios, with positive values representing females being larger than males.

Sexual size dimorphism (in SVL) is uncommon in skinks and within each subfamily (Figure 6). The overall Gibbons and Lovich (1990) ratio, 0.029 ± 0.113 SD, indicates a very slight female‐bias. Out of the 764 species we have data for, females are larger in 450, males in 282 and 32 are identical, but ratios (mean SVL of the larger sex divided by that of the smaller) exceed 10% only in 220 species (in 153 females are larger, in 67 males are larger), with 544 species being nearly monomorphic.

**FIGURE 7:**
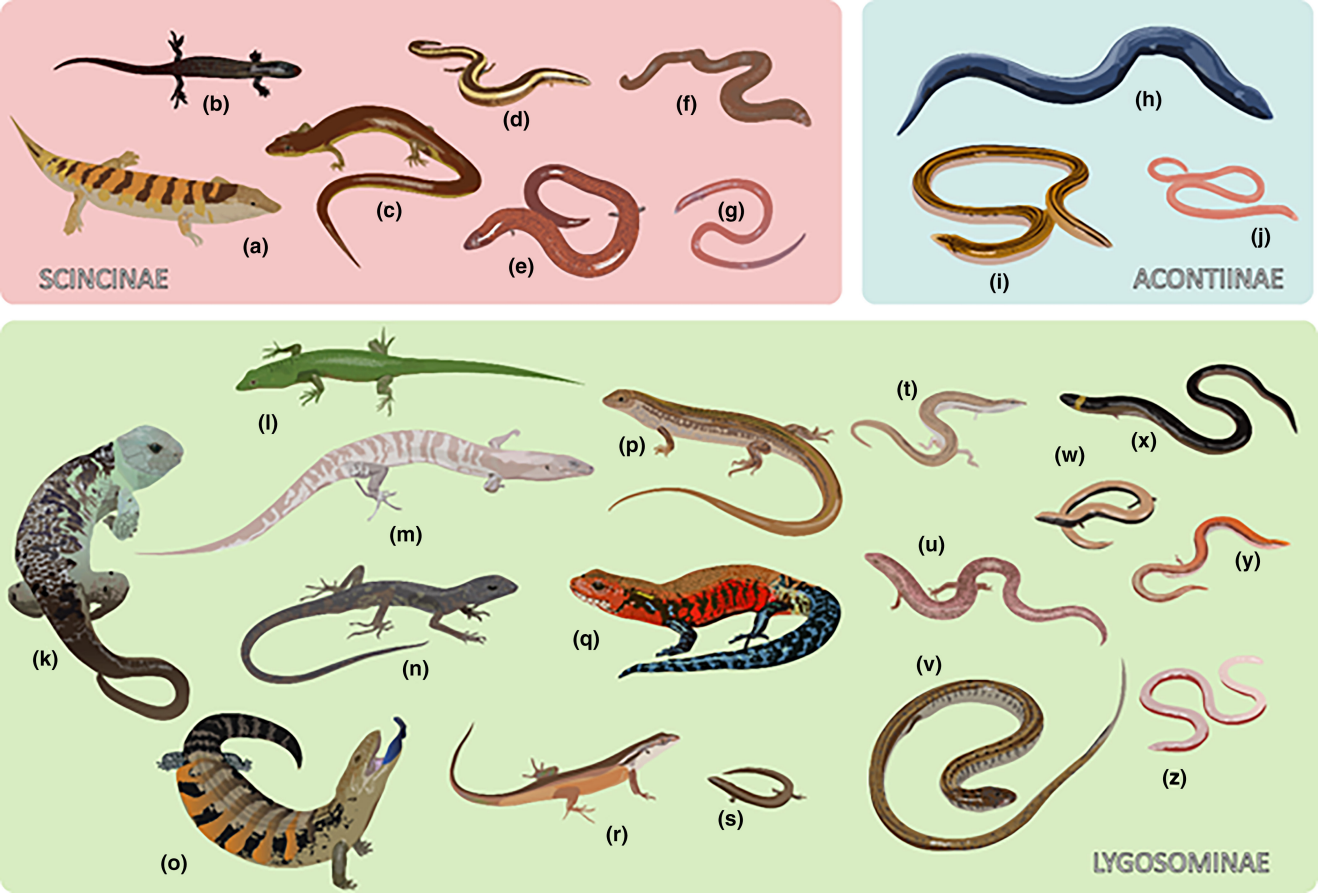
Representative body shape and size variation across the three main subfamilies of skinks (Scincidae). (a) *Scincus scincus*; (b) *Chalcides ocellatus*; (c) *Amphiglossus astrolabi*; (d) *Scelotes limpopoensis*; (e) *Brachymeles elerae*; (f) *Feylinia polylepis*; (g) *Voeltzkowia yamagishii*; (h) *Acontias plumbeus*; (i) *Acontias lineatus*; (j) *Typhlosaurus vermis*; (k) *Corucia zebrata*; (l) *Lamprolepis smaragdina*; (m) *Phoboscincus bocourti*; (n) *Fojia bumui*; (o) *Tiliqua scincoides*; (p) *Ctenotus robustus*; (q) *Mochlus fernandi*; (r) *Carlia longipes*; (s) *Pygmaeascincus timlowi*; (t) *Lerista planiventralis*; (u) *Glaphyromorphus punctulatus*; (v) *Eumecia anchietae*; (w) *Saiphos equalis*; (x) *Anomalopus verreauxii*; (y) *Lerista bipes*; (z) *Lerista apoda*.

Skinks exhibit substantial variation in body shape (Figure 7). Applying Camaiti et al.'s (2022) definition of limb reduction based on limb proportions to SVL, more than one‐fifth (22.8%) of skinks display limb reduction or loss (*n* = 398; 112 limbless; 286 limb‐reduced, of which 4 with forelimbs only, 52 with hindlimbs only), compared with only 3.0% of non‐scincid lizards (*n* = 5288; 108 limbless, 1 forelimbs only, 11 hindlimbs only, 37 limb‐reduced; Figure 7). Across skink subfamilies, all of the Acontiinae (*n* = 32) are limbless, 43.7% (*n* = 129) of the Scincinae are limb‐reduced and 21.7% (*n* = 64) are limbless, but only 10.9% (*n* = 155, of which 115 are in the tribe Sphenomorphini) of the Lygosominae species are limb‐reduced and 1.1% (*n* = 16, all Sphenomorphini) are limbless.

### 3.3 Life history

**FIGURE 8:**
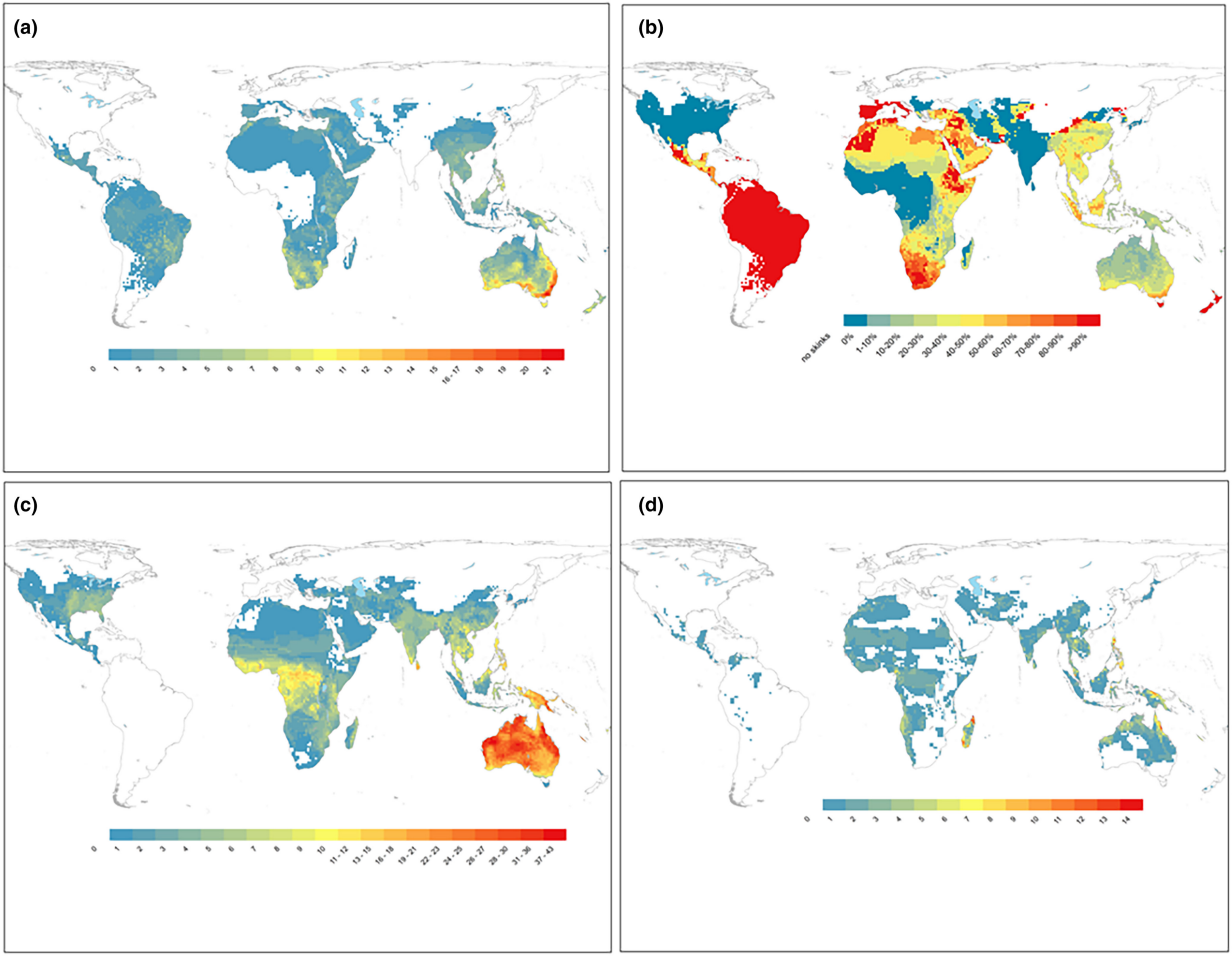
Distribution of reproductive modes in skinks: (a) viviparous species richness, (b) proportion of viviparous species from all species with known reproductive modes, (c) oviparous species richness, (d) richness of species with unknown reproductive mode.

The reproductive mode of 489 skink species (28.0%) is unknown, less than the knowledge gap for non‐scincid squamates (39.4%, *n* = 5012; *χ* ^2^ = 72.4, *p* < .0001). Only including those species with known reproductive modes, 34.3% of skink species are viviparous (vs. 12.6% for non‐scincids), 64.7% are oviparous (vs. 87.2% for non‐scincids), and 1.0% have a mixed reproductive mode (vs. 0.2% for non‐scincids). Thus, skinks are more likely to be viviparous than non‐scincid lizards (*χ* ^2^ = 277.1, *p* < .0001). The percentage of viviparous species is highest in Acontiinae (25 of 26 species with known reproductive mode; 96.2%), lowest in the Lygosominae (27.7%; species with mixed reproductive mode omitted) and intermediate in the Scincinae (65.6%). Viviparous species are more common in southern and alpine Australia, New Zealand, northern Asia, Europe, northern and southern Africa, and central and South America (Figure 8).

**FIGURE 9:**
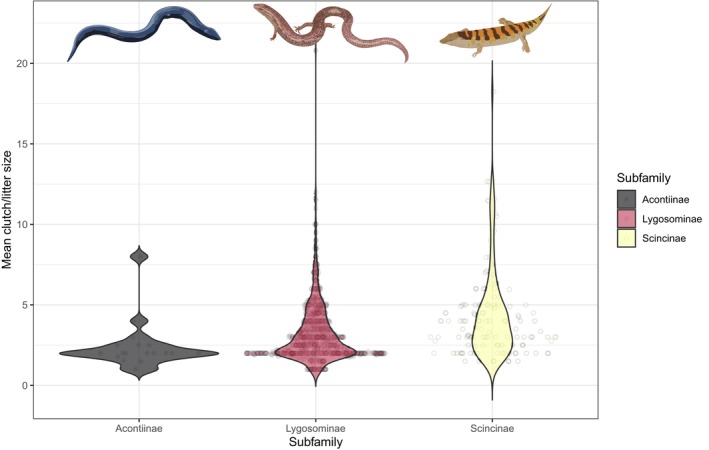
Violin plot comparing clutch/litter sizes among skink (Scincidae) subfamilies.

The mean clutch/litter size of skinks (3.4 ± 2.1; *n* = 926) is lower than that of non‐scincid lizards (3.9 ± 4.4, *n* = 2993; *t* = 2.06, *p* = .039, test run on log_10_‐transformed data). However, after correcting for mass and reproductive mode (log_10_‐transformed brood sizes are higher by 18% for viviparous species and increase with log_10_‐transformed mass with a slope of 0.25), skink broods are actually 14% higher (*t* = 5.87, *p* < .0001). Among skinks the mean clutch/litter size is highest in the Scincinae (4.4 ± 2.9_SD_), lowest in the Acontiinae (2.4 ± 1.5_SD_) and intermediate in the Lygosominae (3.2 ± 1.9_SD_) (Figure 9).

**FIGURE 10:**
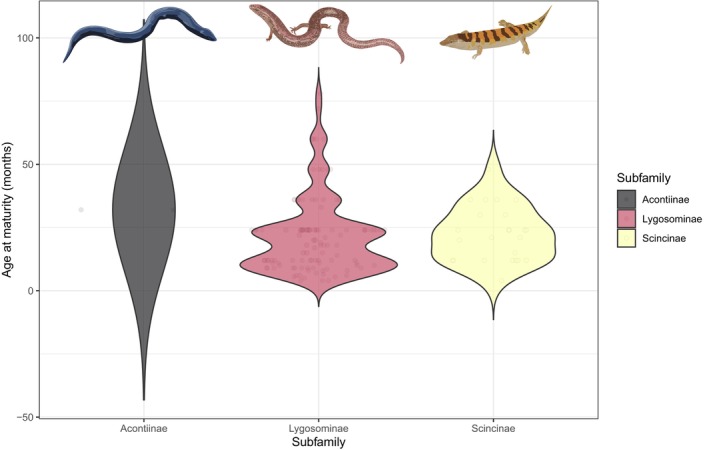
Violin plot comparing age at maturity among skink (Scincidae) subfamilies.

The age at maturity for skinks (25.0 ± 15.8_SD_ months, *n* = 135) is older than that for non‐scincid lizards (20.6 ± 18.0 months, *n* = 585; *t* = 3.89, *p* = .0001, ages log_10_‐transformed, averaged and back transformed). This difference intensifies when body mass (which is positively corrected with age at maturity, with a slope of 0.155, both age and mass being log_10_‐transformed) is accounted for. Acontiine skinks take the longest to mature (32 months, but our sample size is only 2 species), whereas Lygosominae and Scincinae mature at similar ages (25.0 ± 16.9, *n* = 109, and 24.5 ± 16.9 months, *n* = 24, respectively; Figure 10).

**FIGURE 11:**
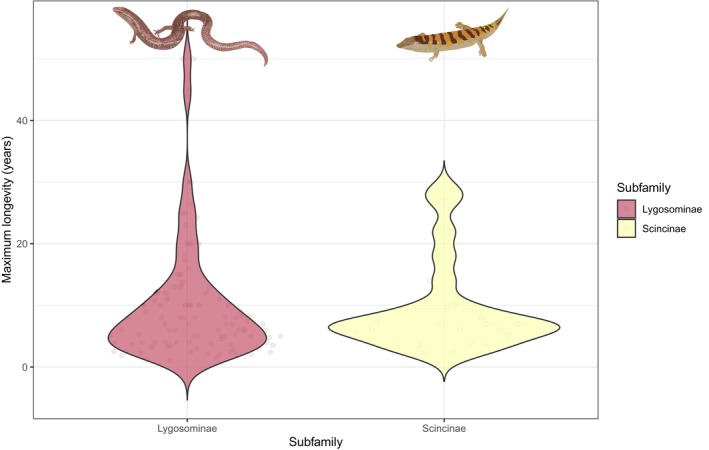
Violin plot comparing maximum longevity among skink (Scincidae) subfamilies.

Maximum skink longevity (10.0 ± 9.4 years, *n* = 130) is similar to that of other lizards (10.6 ± 10.0 years, *n* = 688) regardless of whether mass is corrected for (*t* = 0.32, *p* = .75, mass and longevity log_10_‐transformed) or not (*t* = 0.63, *p* = .53). Within skinks, differences between lygosomines (mean maximum longevity 10.1 ± 9.9, *n* = 106) and scincines (9.5 ± 7.5 years, *n* = 23) are small and not statistically significant whether we correct for mass (*t* = 0.55, *p* = .58) or not (*t* = 0.25, *p* = .81) (Figure 11). The single acontiine for which we have a longevity datum is *Acontias meleagris*, with a maximum recorded longevity of 3.9 years.

### 3.4 Ecology

**FIGURE 12:**
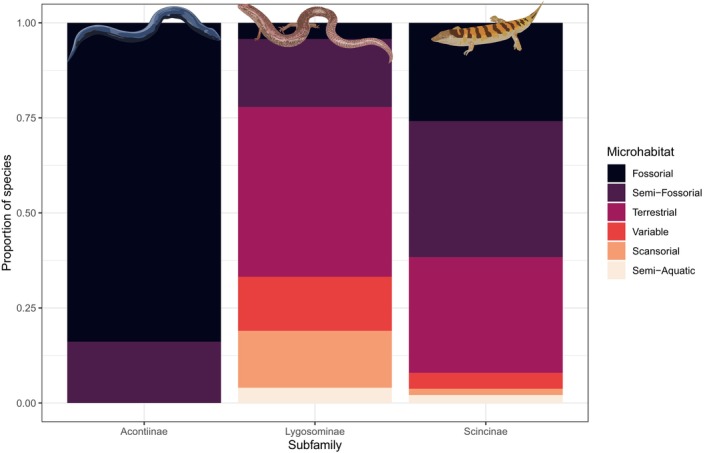
Microhabitats of skink subfamilies.

Microhabitat varies substantially among skink subfamilies (Figure 12). All acontiines are fossorial or semi‐fossorial, as are 62% of the species in the Scincinae (in which 30% of the species are terrestrial) whereas only 22% of species in the Lygosominae are full or partially fossorial. Lygosomines are more varied with 45% of the species being terrestrial, 15% scansorial (climbing trees and/or rocks), 14% frequenting varied microhabitats (both scansorial and terrestrial), and 4% are semi‐aquatic (Figure 12).

**FIGURE 13:**
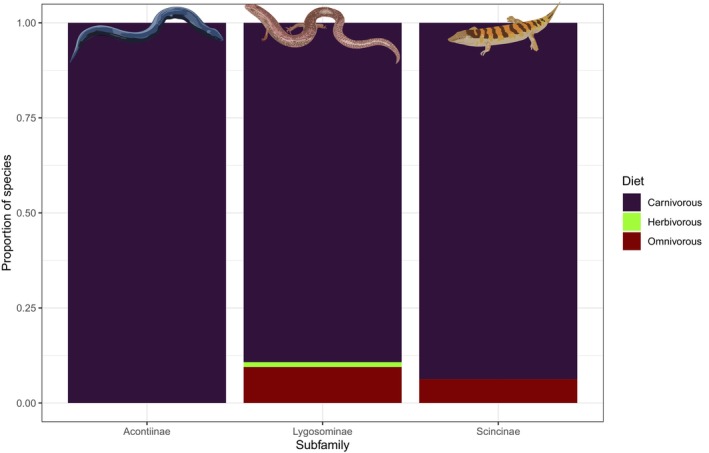
Diet of skink subfamilies.

The diet of 814 skink species (46.7%) is unknown, which is comparable to the knowledge status for non‐scincids (47.5%, 2382 of 5012 species). Of these 814 skink species, 90.1% are carnivorous, 8.8% are omnivorous, and 1.1% are herbivorous (vs. 77.3%, 17.0% and 5.7% for non‐scincids, respectively; *χ* ^2^ = 77.23, *p* < .0001). Members of the Acontiinae are all carnivorous, while 6.2% of scincine species with known diet are omnivorous (the others are carnivorous). In the Lygosominae, 9.5% of species with known diets are omnivores and 1.2% (*n* = 10) feed mostly on plants (Figure 13).

Data on the foraging mode of skinks are only available for 223 species, of which 87.4% are active foragers, while 6.3% are sit‐and‐wait predators, and 6.3% have a mixed foraging mode.

**FIGURE 14:**
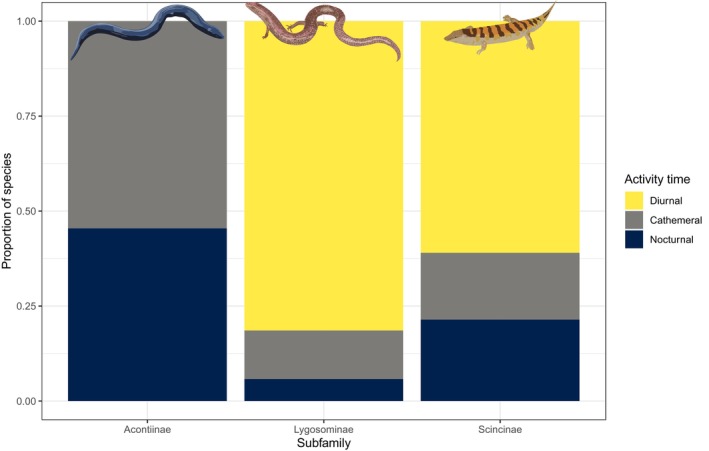
Activity times proportion (diurnal: yellow, cathemeral: light blue, nocturnal: black) of skink subfamilies.

The activity pattern of 23.4% of skink species is unknown, considerably more than in non‐scincid lizards (19.2%; *χ* ^2^ = 14.2, *p* = .0002). For the species where activity patterns are known, skinks have a higher incidence of diurnal (78.0% vs. 67.9%) and cathemeral (13.8% vs. 4.3%) species compared with non‐scincid lizards. In contrast, there are relatively few nocturnal skinks (8.2%) compared to nocturnal non‐scincid lizards (27.7%; *χ* ^2^ = 313.4, *p* < .0001). Acontiinae is the most unusual skink subfamily in terms of activity pattern, with all species either nocturnal (5) or cathemeral (6) (Figure 14). Indeed, its lack of diurnal species is striking, particularly given that the majority of species in the Scincinae (61%) and Lygosominae (81%) are diurnal (Figure 14).

The mean field body temperature (*T*_b_) of skinks (mean *T*_b_: 30.5 ± 4.1°C, *n* = 195 species) is slightly lower than that of non‐scincid lizards (mean *T*_b_: 31.4 ± 4.9°C, *n* = 1015; *t* = 2.03, *p* = .02). When activity times are taken into account, diurnal skinks are active at slightly lower body temperatures (by 1.3°C, on average) than non‐scincid lizards, but nocturnal and cathemeral skink body temperatures are higher than those of other lizards with similar activity times (Table S1). The single acontiine for which we have data on body temperature (*Acontias meleagris*) is active at a low 21.8°C. There is relatively little difference between Scincinae (mean 30.1°C ± 3.4_SD_, *n* = 29) and Lygosominae (mean 30.7°C ± 4.2_SD_, *n* = 165) in their field body temperature: these are not statistically significantly different whether activity times are taken into account (*t* = 0.65, *p* = .52) or not (*t* = 0.72, *p* = .48; there is no activity time:subfamily interaction, *p* = .83). Preferred body temperatures in a thermal gradient are positively correlated with field body temperatures (*n* = 73 species with both temperature indices, *R* ^2^ = .53); however, the slope (0.74 ± 0.08) is significantly shallower than 1, suggesting that skinks preferring cold temperatures may have difficulty getting warm enough, while species preferring warm temperatures can barely keep cold enough. However, the model predicts that body temperatures will equal preferred temperatures at 30.64°C – remarkably close to the 30.58°C average *T*_b_ for active skinks as a whole.

## 4 DISCUSSION

Our study has demonstrated that skinks are typically small lizards (in terms of both SVL and mass), lack sexual dimorphism, are active foraging carnivores and exhibit diurnal or cathemeral activity patterns. Skink species generally have larger geographic ranges than other lizards and display both areas of high species diversity (Australia, New Guinea, southeast Asia, Oceania, Madagascar, southern and central Africa) and species paucity (the Americas, Europe). Ironically, although the standard skink body plan appears to have been highly successful, part of the success of skinks lies in their ability to frequently deviate from their typical body plan, with repeated transitions from oviparity to viviparity and from fully limbed species to limb‐reduced or limbless species. The three skink subfamilies are inconsistent in their expression of these distributional, morphological, life history and ecological attributes, with the Acontiinae deviating substantially from most of the broader trends in skinks.

### 4.1 Skinks: a diverse and rapidly growing reptile group

Skinks currently comprise almost a quarter (24%) of the known lizard fauna globally, and as approximately 20 new species are described each year (Figure 1), this number is rapidly growing (Uetz et al., 2022). However, while 16% of new lizard species described since 2010 have been skinks, this is substantially less than the growth of gekkotans which, over the same period, have accounted for almost half (44%) of lizard descriptions (Meiri, 2016, 2019; Uetz et al., 2020). We found that skinks currently have geographic ranges that are larger than non‐scincid lizards; however, this pattern may be eroded if the current rate of species description continues. This is because more recently described lizard species have smaller distributions (Meiri, 2016). In addition, partly as a consequence of having smaller geographic ranges, more recently described species are more likely to be threatened (Caetano et al., 2022; Meiri, 2016). This is particularly a problem as >85% of reptile species are listed on the IUCN Red List under Criterion B, which relates to geographic range size (Chapple et al., 2021; Cox et al., 2022; Meiri et al., 2023). Alternatively, it is possible that the broader geographic ranges evident for skinks could partly be due to some widespread species representing unrecognised species complexes (e.g. Chapple et al., 2021; Melville et al., 2021), and future taxonomic work that splits these complexes into multiple taxa, may lead to an increase in species diversity, but also a decrease in geographic range size in skinks.

Although we highlight that skinks have a cosmopolitan distribution (apart from Antarctica), their centres of diversity are in Australia, New Guinea, southeast Asia, Oceania, Madagascar and southern and central Africa. Apart from the hotspot in Oceania and New Guinea, which are disproportionately dominated by skinks (Slavenko et al., 2023), these diversity hotspots are largely shared with the Gekkota superfamily (Meiri, 2020), but are strikingly different from those evident in the most speciose reptile family, the colubrids (O'Shea, 2011, 2023). Colubrids are widespread throughout North America and Europe, and occur throughout most regions of the world, but are absent from the majority of Australia (O'Shea, 2011, 2023; Shine, 1991). The reasons for the relative paucity of skinks in the New World are poorly studied. Most skinks in North America, central America and Europe are in the subfamily Scincinae (Figure 2). Members of the genus *Plestiodon* are thought to have been present in North America for around 18–30 million years (Brandley et al., 2012), without achieving significant diversity or colonising South America. In addition, it is also unclear why members of the more successful Lygosominae have not speciated and spread more throughout the Americas.

### 4.2 Skinks have small body size and exhibit frequent transitions to limb reduction or loss

Our study demonstrates that skinks typically have small body sizes, both in terms of SVL and mass.

Within squamates more broadly, body size variation reaches up to six orders of magnitude (Feldman et al., 2016); however, as skinks are better represented in the smallest lizard species globally (6th and 10th smallest), than the largest lizard species (74th, 77th, 99th largest), the degree of body size variation within the family is relatively lower. Specifically, the largest limbed skink species *Bellatorias major* is 6404 times heavier, and 16 times longer (in SVL), than the smallest known limbed skink, *Scincella macrotis*. Unlike many endothermic lineages, squamate body size does not appear to be driven by climatic factors (Slavenko et al., 2019), and may be more shaped by species‐specific ecology or habitat requirements. For instance, members of the subfamily Acontiinae are highly specialised for a fossorial lifestyle (Camaiti et al., 2022), and are characterised by being extremely long, but relatively lighter, compared with limbed species of an equivalent length. Although sexual size dimorphism is common within reptiles (Cox et al., 2003; Scharf & Meiri, 2013), we found that most skinks species are relatively monomorphic. In squamates, where sexual size dimorphism is present, it is generally males that have larger body size (Liang et al., 2022). But in contrast, we found that in skink species where substantial dimorphism was present (i.e. >10% difference in SVL), females had longer SVL on ~70% of occasions.

Our study indicates that ~23% of skink species exhibit some degree limb reduction or loss. Skinks exhibit substantial variation in body shape, displaying a high diversity of forms and body sizes (Figure 7), going from stocky crevice‐dwelling forms characterised by large, laterally expanded heads and bodies and short but powerful legs, to long‐legged, agile arboreal forms, to legless, small‐headed forms with streamlined, cylindrical bodies (what we like to term the ‘kebabs to noodles continuum’) (Camaiti et al., 2022). Indeed, skinks display more deviations from their standard body plan compared with all other lizard families (Camaiti et al., 2021). Perhaps the most prominent and evolutionarily successful example of dramatic body shape modifications appearing in all skink subfamilies is limb reduction. This morphological transformation involves the reduction in both the size and number of elements of the limbs and is often paired with the elongation of the trunk, changes which evolve as adaptations to locomoting more efficiently within or in close contact with complex three‐dimensional mediums like the substrate (Camaiti et al., 2019, 2021). Not only is this type of morphological adaptation common, but it is thought to have evolved independently between 53 and 71 times (Camaiti et al., 2022), in all continents except Antarctica and South America. For instance, while all members of the Acontiinae subfamily are limbless, limb reduction (44% Scincinae, 11% Lygosominae) and complete limb loss (22% Scincinae, 1% Lygosominae) is common in the other two skink subfamilies.

### 4.3 A high incidence of viviparity and ‘slow’ life histories in skinks

Around a third of skink species are viviparous, and this reproductive mode is significantly more prevalent in skinks compared with other lizards. Viviparity has had more independent origins in squamates (>100 times) than any other vertebrate group, and this has been largely driven by skinks, which alone account for at least 31 transitions from oviparity to viviparity (Blackburn, 1982, 1999, 2015). In squamates, viviparity is more common in cold climates (Zimin et al., 2022), and therefore the prevalence of this reproductive mode in skinks may have assisted the group to reach high diversity (compared to other lizard groups) at high latitudes and high elevations (see Figures 2 and 8). After accounting for body size, the clutch sizes of oviparous species are equivalent to the litter sizes for viviparous species (Meiri, Feldman, et al., 2020). Interestingly, we found that skinks (after adjusting for body size and reproductive mode) have larger clutch/litter sizes than non‐scincid lizards. Lizard clutch/litter sizes are generally larger at higher latitudes and in seasonal environments (e.g. deserts) (Meiri, Avila, et al., 2020), which are regions that skinks have high density (see Figure 2). Thus, skinks are lizards that are characterised by high rates of viviparity and relatively large clutch/litter sizes.

Our results indicate that skinks mature later, but have similar lifespans, to other lizard groups. Skinks generally have smaller body sizes than other lizards; however, although life span in squamates is linked to body size, it only explains a relatively small portion of the variation (Scharf et al., 2015; Stark et al., 2018), and therefore could explain why skinks are able to achieve similar longevity to other lizards. In squamates, there is generally a strong correlation between age at maturity and lifespan (Scharf et al., 2015). Thus, it is interesting that skinks reach maturity later than other lizards but have similar lifespans. Squamates at higher latitudes, and in cold regions, generally take longer to reach maturity (Stark et al., 2018)—a result that is thought to be due to the shorter activity season in these regions, resulting in slower development and later maturity (Scharf et al., 2015). The global distribution of skinks (Figure 2), and the high rate of viviparity in the group, demonstrate that skinks are prevalent in relatively cold regions, which could result in slower development and later maturity of skink species.

### 4.4 The stereotypical skink is a diurnal, active foraging carnivore

Our results indicate that skinks are generally diurnal, active foraging carnivores. In lizards, diet is closely associated with body size, with omnivorous and herbivorous species tending to have larger body sizes than carnivorous species (Chapple, 2003; Espinoza et al., 2004; Meiri, 2008; van Damme, 1999). Thus, the smaller body size of skinks may explain why skinks are more likely to be carnivorous compared with other lizards. Although the diet of around half of skink species is unknown, given the strong relationship between SVL and diet (Chapple, 2003), body size could be used to predict the likely dietary mode of skink species currently lacking data. Similarly, smaller lizard species are more likely to be diurnal (Meiri, 2008), a result that is also evident to some extent in skinks (Slavenko et al., 2022). For instance, although a quarter of lizard species worldwide are nocturnal, which is the predominate activity mode for gekkotans (Meiri, 2020), only 8% of skink species are nocturnal. However, nocturnal lizards are largely absent from high elevations and cold climates (Vidan et al., 2017), and skinks exhibit relatively high diversity in these regions. Indeed, nocturnality in skinks in associated with fossoriality, limb reduction and loss and inhabiting warmer temperatures (Slavenko et al., 2022). Interestingly, even after taking activity mode into account, skinks appear to be active at lower body temperatures than other lizard species. Intriguingly, however, cathemeral and nocturnal skinks exhibit the opposite trend and are active at higher body temperatures compare to other lizard groups.

## 5 CONCLUSIONS

Are skinks, and other diverse groups (e.g. gekkotans, colubrids), successful because they are uniform in their morphology, ecology and life history or are they successful because they exhibit variation in key traits? Our study indicates that skinks are on the one hand uniform—exhibiting a great propensity for small body size (and being sexually monomorphic in body size), diurnality, active foraging and carnivory. But despite this tendency for uniformity, skinks are the poster child for key evolutionary transitions in limb reduction and the evolution of viviparity. As a lineage, skinks appear to be evolutionarily ‘malleable’, filling every available ecological niche by either staying as close as possible to their standard body plan and ecology (which appears to be a good design that works the majority of times) or evolving rapidly in different directions to exploit extreme environments—that is limb reduction and loss in fossorial environments (Camaiti et al., 2023), and viviparity in cold climates (Zimin et al., 2022). Thus, skinks appear to be the ‘Jack of All Trades’ of squamates, while retaining the potential to change in certain situations and environments.

## AUTHOR CONTRIBUTIONS

**David G. Chapple:** Conceptualization (equal); data curation (equal); funding acquisition (lead); investigation (equal); methodology (equal); project administration (equal); writing – original draft (equal). **Alex Slavenko:** Data curation (equal); formal analysis (equal); visualization (equal); writing – review and editing (equal). **Reid Tingley:** Conceptualization (equal); formal analysis (equal); methodology (equal); visualization (equal); writing – review and editing (equal). **Jules E. Farquhar:** Data curation (equal); formal analysis (equal); visualization (equal); writing – review and editing (equal). **Marco Camaiti:** Data curation (equal); formal analysis (equal); methodology (equal); visualization (equal); writing – review and editing (equal). **Uri Roll:** Data curation (equal); formal analysis (equal); methodology (equal); visualization (equal); writing – review and editing (equal). **Shai Meiri:** Conceptualization (equal); data curation (equal); formal analysis (equal); funding acquisition (equal); investigation (equal); methodology (equal); visualization (equal); writing – original draft (equal).

## CONFLICT OF INTEREST STATEMENT

The authors declare no conflicts of interest.

## Supporting information


Appendix S1

Appendix S2

Data S1

## Data Availability

Our data are provided in the supplementary material (Appendices S1 and S2).
