# Supplementary material for: Built for success: Distribution, morphology, ecology and life history of the world's skinks
Source: Ecol Evol. 2023 Dec 12;13(12):e10791. doi: 10.1002/ece3.10791 (PMC10716605; doi:10.1002/ece3.10791)
Supplement: Supplementary file 1 — Appendix S1 [file ECE3-13-e10791-s002.zip › Appendix_1_-_skink_Biology.docx]

Here is the caption for Appendix 1:

Summary traits for skinks globally that were used in this study. The dataset has been updated from Meiri (2018) [Meiri S (2018) Traits of lizards of the world: variations around a successful evolutionary design. Global Ecology and Biogeography 27: 1168-1172.].
