# Supplementary material for: Built for success: Distribution, morphology, ecology and life history of the world's skinks
Source: Ecol Evol. 2023 Dec 12;13(12):e10791. doi: 10.1002/ece3.10791 (PMC10716605; doi:10.1002/ece3.10791)
Supplement: Supplementary file 2 — Appendix S2 [file ECE3-13-e10791-s001.zip › Appendix_2-_skink_lengths_and_masses.docx]

Here is the caption for Appendix 2:

Skink data on body size (snout-vent length, mass) used in this study. The references for the collated data are provided.
