# Supplementary material for: Built for success: Distribution, morphology, ecology and life history of the world's skinks
Source: Ecol Evol. 2023 Dec 12;13(12):e10791. doi: 10.1002/ece3.10791 (PMC10716605; doi:10.1002/ece3.10791)
Supplement: Supplementary file 3 — Data S1 [file ECE3-13-e10791-s003.docx]

**Supplementary Material for:**

**Built for Success: distribution, morphology, ecology and life history of the world’s skinks**

**Figure S1.** Relationship between snout-vent length (SVL; log SVL) and body mass (log weight) in skinks and skink subfamilies. We considered limbed, limb reduced and limbless species separately.

**Table S1.** Mean field body temperature (Tb) of skinks and non-skinks, taking into account their activity mode.

|  |  | n | mean tb | tb sd |
| --- | --- | --- | --- | --- |
| skinks | Diurnal | 156 | 31.0 | 3.6 |
|  | Nocturnal | 8 | 27.7 | 2.7 |
|  | Cathemeral | 31 | 28.7 | 5.8 |
| non-skinks | Diurnal | 866 | 32.3 | 4.3 |
|  | Nocturnal | 103 | 25.6 | 4.7 |
|  | Cathemeral | 46 | 27.0 | 4.1 |
